# Varisized 3D‐Printed Lunate for Kienböck's Disease in Different Stages: Preliminary Results

**DOI:** 10.1111/os.12681

**Published:** 2020-05-17

**Authors:** Zhen‐jiang Ma, Zi‐fan Liu, Qing‐song Shi, Tao Li, Zhi‐yuan Liu, Ze‐zheng Yang, Yi‐hao Liu, Yuan‐jin Xu, Kerong Dai, Chao Yu, Yao‐kai Gan, Jin‐wu Wang

**Affiliations:** ^1^ Shanghai Key Laboratory of Orthopaedic Implant, Department of Orthopaedic Surgery Shanghai Ninth People's Hospital, Shanghai Jiao Tong University School of Medicine Shanghai China; ^2^ College of Medicine Southwest Jiao Tong University Sichuan China; ^3^ Department of Orthopaedic Surgery Yuyao People's Hospital of Zhejiang Province Yuyao China; ^4^ School of Biomedical Engineering Shanghai Jiao Tong University Shanghai China

**Keywords:** 3D printing, Kienböck's Disease, personalized prosthesis

## Abstract

**Objective:**

To evaluate the feasibility of arthroplasty with varisized three‐dimensional(3D) printing lunate prosthesis for the treatment of advanced Kienböck's disease (KD).

**Methods:**

From 2016 November to 2018 September, a retrospective study was performed for the patients of KD in our hospital. Five patients (two males, three females) were included in this study. The mean age of the patients at the time of surgery was 51.6 years (range, 37–64 years). Varisized prosthesis identical to the live model in a ratio of 1:0.85, 1:1, and 1:1.1 were fabricated by 3D printing. All patients (one in Lichtman IIIA stage, two in Lichtman IIIB stage, one in Lichtman IIIC stage, and one in Lichtman IV stage) were treated with lunate excision and 3D printing prosthetic arthroplasty. Visual analog scale score (VAS), the active movement of wrist (extension, flexion) and strength were assessed preoperatively and postoperatively. The Mayo Modified Wrist Score (MMWS), Disabilities of the Arm, Shoulder and Hand (DASH) Score, and patient's satisfaction were evaluated during the follow‐up.

**Results:**

Prosthesis identical to the live model in a ratio of 1:0.85 or 1:1 were chosen for arthroplasty. The mean operation time (range, 45 to 56 min) was 51.8 ± 4.44 min. Follow‐up time ranged from 11 months to 33 months with the mean value of 19.4 months. The mean extension range of the wrist significantly increased from preoperative 44° ± 9.6° to postoperative 60° ± 3.5° (*P* < 0.05). The mean flexion range of the wrist significantly increased from preoperative 40° ± 10.6° to postoperative 51° ± 6.5° (*P* < 0.05). The active movement of wrist and strength were improved significantly in all patients. VAS was significantly reduced from 7.3 preoperatively to 0.2 at the follow‐up visit (*P* < 0.05). The mean DASH score was 10 (range, 7.2–14.2), and the mean MMWS was 79 (range, 70–90). There were no incision infection. All patients were satisfied with the treatment.

**Conclusions:**

For patients suffering advanced Kienböck's disease, lunate excision followed by 3D printing prosthetic arthroplasty can reconstruct the anatomical structure of the carpal tunnel, alleviate pain, and improve wrist movement.

## Introduction

Ischemic necrosis of lunar bone, namely Kienböck's disease (KD), was initially described by a Viennese radiologist (Robert Kienböck) in 1910. Since KD was first described, numerous surgical treatments have been proposed for patients unresponsive to nonoperative treatment^1^, ^2^, ^3^, ^4^. These surgical options can be classified into lunate unloading procedures, lunate revascularization, salvage procedures, and replacement procedures^5^. Despite the various alternatives, the surgical treatment of KD remains controversial and no consensus has been reached on the optimal treatment since none of these procedures have been fully successful in reconstructing and maintaining the height of the carpal tunnel, avoiding progression of the disease, restoring the full function of the wrist, and alleviating the pain^5^, ^6^, ^7^. Furthermore, the published literature on KD only focused on the functional results, whereas the achievement of the anatomical objective which is vital to the prognosis is often omitted. These happen even in studies detailing techniques that aim to revascularize the lunate or help it regain its original shape^7^. Several studies reported lunate replacement for advanced KD, such as vitallium, acrylic materials and pyrocarbon lunate arthroplasty^8^, ^9^, ^10^. However, these techniques have not been widely applied because of the incongruity and inherent instability of the prostheses^5^.

Three‐dimensional (3D) printing, also termed as additive manufacturing, refers to a process wherein a 3D object is manufactured based on a digital model by means of successive layering of materials following the direction of a computer^11^. Theoretically, this process can produce a prosthesis of any shape with ideal congruity or surface modification allowing precise matching, stable fixation, and restoration of normal loading^11^, ^12^. Following these unique features, personalized 3D‐printed prostheses are hypothesized as an ideal alternative for the replacement of injured bone, and have been increasingly applied to clinical bone defect reconstruction^11^, ^13^. However, the clinical significance of the 3D printing lunate prosthesis in treating different staged KD still remains unknown. Furthermore, there is little information about the specific design and surgical techniques regarding 3D‐printed lunate prosthesis. In order to assess the feasibility of using this technology in manufacturing prostheses for the treatment of advanced KD, a retrospective study was performed with the clinical data of patients who underwent lunate excision and reconstruction using an appropriate 3D‐printing lunate prosthesis.

## Materials and Methods

### *Subjects*

This study was approved by the Committee on Clinical Investigation of our hospital. From November 2016 to September 2018, a retrospective chart review was performed for the patients who underwent lunate replacement arthroplasty with a 3D printing lunate for treatment of KD in our hospital.

Indications for lunate replacement arthroplasty were the patients who suffered KD of Lichtman stage III or stage IV with: (i) continued pain after conservative care; (ii) incapability to fulfill employment because of wrist symptoms; or (iii) unwillingness to pursue further conservative care. Surgical contraindications included: (i) history of septic arthritis; (ii) psychiatric illness; and (iii) systemic illness or condition making surgical intervention hazardous.

**Figure 1:**
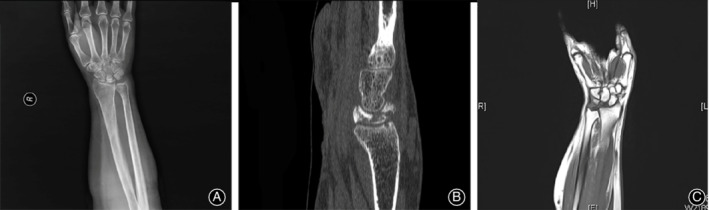
Preoperative radiographic views of the necrotic lunate with Lichtman classification IIIC. (A) X‐ray; (B) Computerized tomographic scanning; (C) Magnetic resonance image.

Five patients were included in this study. The demographic information of patients is listed in Table 1. The mean age of the patients at the time of surgery was 51.6 years (range, 37–64 years). All patients had an occupational or recreational background of repeated minor stress to the wrist and these activities were interrupted by severe wrist pain. The symptoms in the wrist had been present for a mean of 11.4 months (range, 1 to 24). Preoperative radiographic findings were obtained (Fig. 1) and the Lichtman classification was used as the radiologic osseous classification system (Table 1).

**Table 1:** General information of the patients

Case	Age (Yr)	Gender	Side	Symptom duration (Mo)	Lichtman staging	Bain/Begg arthroscopic classification	Operation time (Min)	Follow‐up (Mo)
1	37	M	R	2	IIIA	Grade 3	53	33
2	64	F	R	24	IV	Grade 4	50	22
3	53	M	R	1	IIIB	Grade 2a	56	15
4	58	F	R	6	IIIB	Grade 2a	45	16
5	46	F	R	24	IIIC	Grade 2b	55	11
Mean	51.6			11.4			51.8	19.4

Yr, year; M male; F, female; Min, minute; Mo, month

### *Personalized Lunate Prosthesis Design*

**Figure 2:**
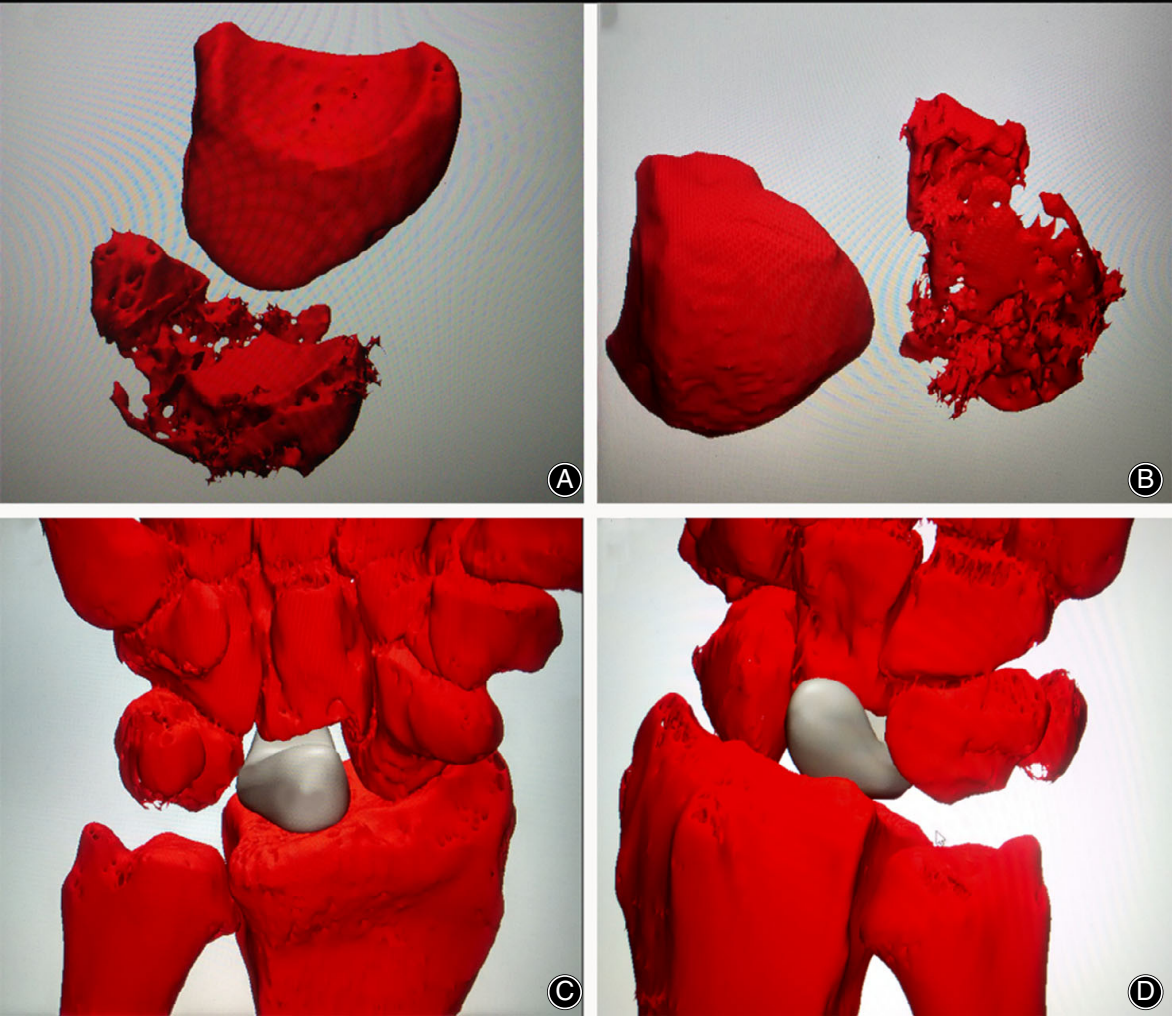
(A and B) show the digital reconstructed images of the injured and opposite lunate from the patient in Fig. 1. (C and D) show the simulation of arthroplasty of the 3D printing lunate in software.

**Figure 3:**
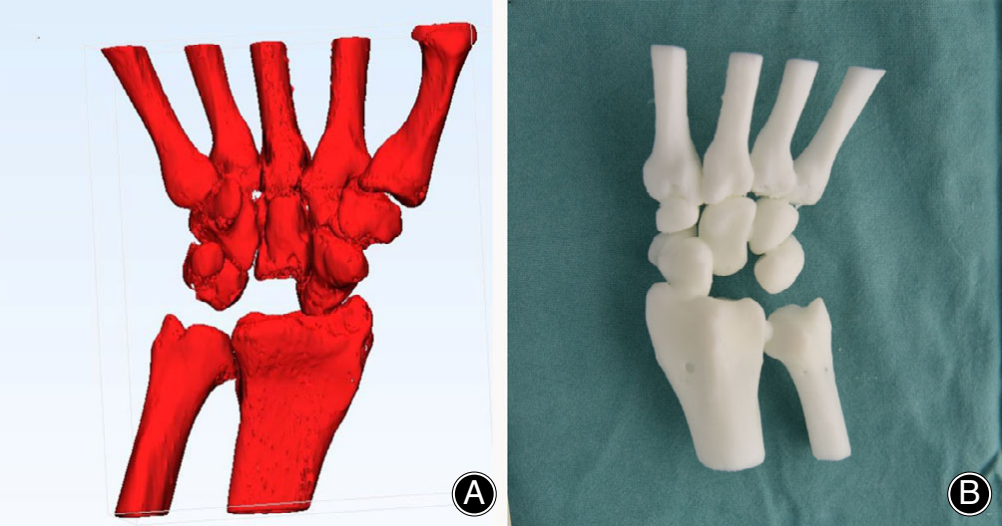
The 3D‐printed wrist model (A, B) made of photosensitive resin with the necrotic lunate removed.

3D prototypes were constructed and manufactured by the doctor in cooperation with the design and manufacturing industry engineer through specialized software and rapid‐prototyping techniques. Briefly, computed tomography (CT) and magnetic resonance imaging (MRI) scans of the bilateral wrists of the patients, taken at 1‐mm intervals, were converted to 3D digital models using image software (Mimics 17.0 and 3‐matic 10.0; Materialize, Leuven, Belgium) (Fig. 2A,B). Afterwards, the digital model was saved and entered into a 3D printing machine. The injured wrist was 3D‐printed at a 1:1 scale using photosensitive resin with the necrotic lunate removed from the wrist for later matching (Fig. 3).

**Figure 4:**
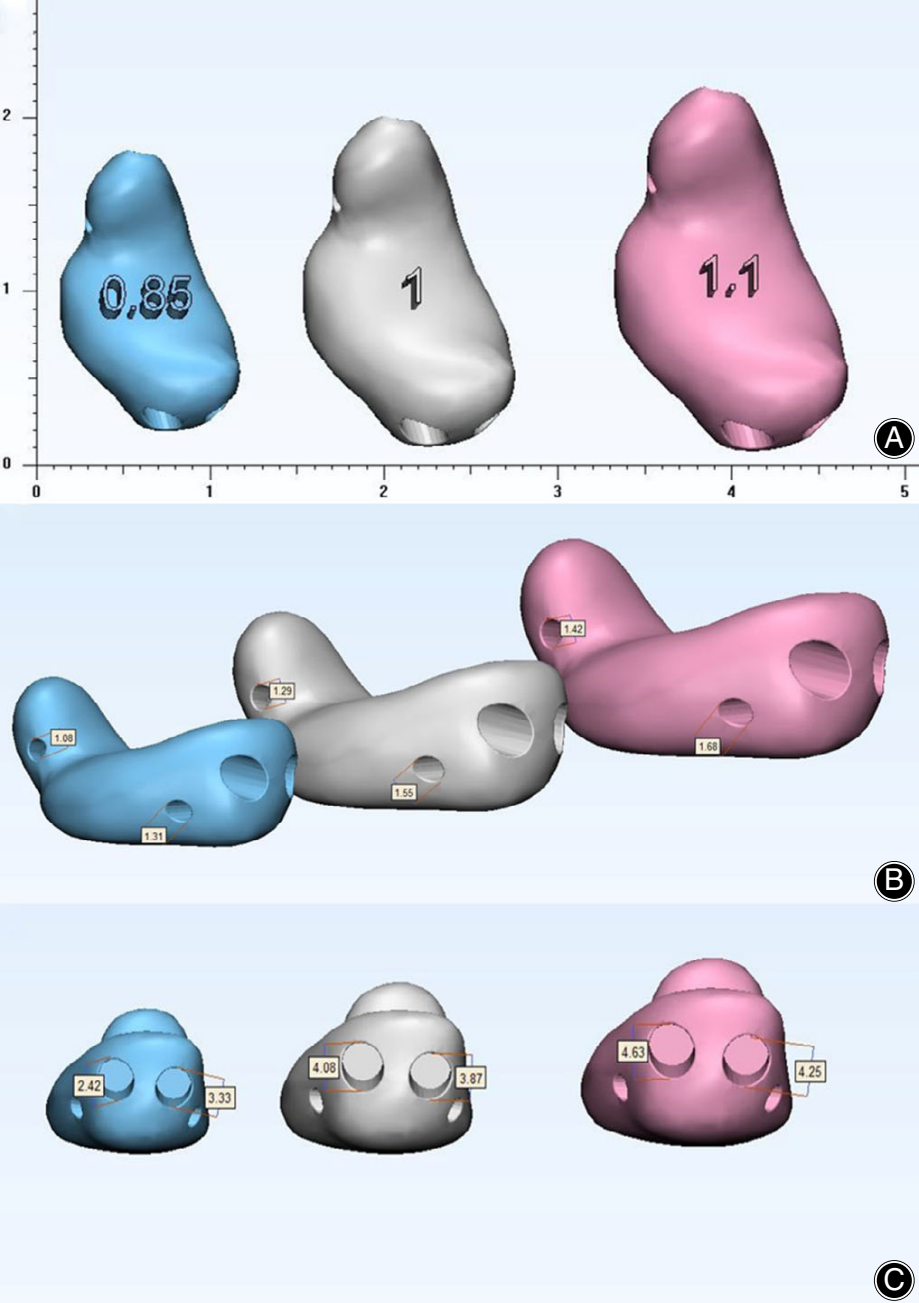
The design of 3D‐printing lunate in three different scales and modifications. (A) view of distal articular surface of lunate; (B) view of lateral side of lunate; (C) view of the palmar side of lunate.

The injured lunate was subsequently designed using the mirror image of the 3D information of the impaired lunate to ensure the same morphological characters. The original anatomical model was modified in three aspects (Fig. 4). First, two transverse tunnels (1‐mm in diameter) across the distal part of the prosthesis were created to permit the passage of the sutures and facilitate the fixation of the prosthesis. Second, if necessary, two inclined channels (proximal: 4‐mm in diameter; distal: 1‐mm in diameter) located at the proximal head of the lunate were also designed for fixation. In addition, the large proximal holes could be used for clamping and manipulation using the hemostatic forceps. Third, to insert the prosthesis anatomically and prevent subluxation, we fabricated varisized prosthesis identical to the live model in a ratio of 1:0.85, 1:1, and 1:1.1. Preoperative surgical simulation with the 3D‐printed photosensitive resin models was performed to check the accuracy of the printing lunate.

### *Manufacture of the Lunate*

During the preoperative surgical simulation, the lunate prosthesis matched the wrist precisely. The final prosthesis, made of titanium alloy (Ti‐6Al‐4V) by means of electron beam melting technology, was fabricated by the company (Thytec Shanghai Co., Ltd., China) with the certification by the China Food and Drug Administration^12^. During the process of rapid‐prototyping, structures such as holes and tunnels were created on the surface of the lunate. After post‐processing, the lunate was ready for use.

### *Surgery*

All procedures were performed by the same surgeon, and the specific process was listed in the following five steps.

#### *Step 1: Anesthesia and Position*

After general anesthesia or brachial plexus block, the patients were maintained at supine position in a standard operating table.

#### *Step 2: Approach and Exposure*

After completed skin preparation and draping*,* the injured arm was elevated and exsanguinated from the fingers down for visualization of the vessels. Then, a 5‐cm “S” incision was made on the dorsal of the wrist with sharp dissection down to the retinaculum. During the process, special attention was paid in identifying and protecting the sensory branch of the radial and ulnar nerves. Then, a longitudinal incision (4–5 cm) was made between the third and fourth compartments of the extensor retinaculum. Dorsal joint capsule was exposed and opened to approach the radiocarpal joint. The lunate was also exposed.

#### *Step 3: Pathological Changes and Resection*

**Figure 5:**
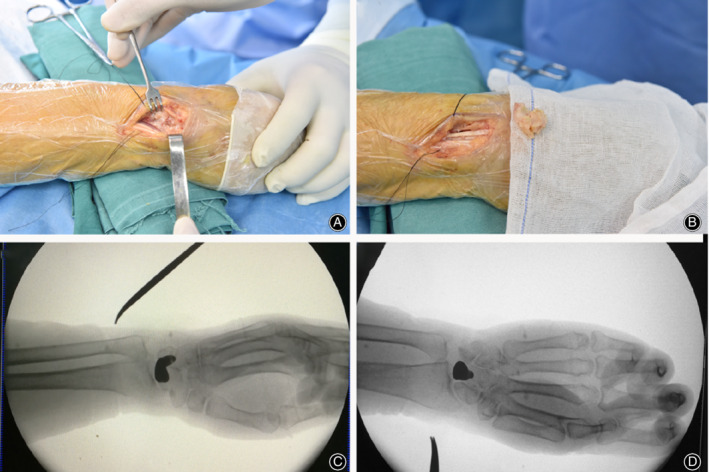
(A and B) show the intra‐operative view of necrotic lunate in position and after removal. (C and D) show the anterior and lateral view of the 3D printing lunate after insertion.

Some pathological changes were observed as follows: yellowish lunate, lunate bone mass decrease and fragility, carpal collapse, cystic degeneration, hyperplasia of local synovial membrane, etc. Lunate volar dislocation and adhesion with surrounding carpus were also observed in some patients. Local lunate gap was filled with granulation tissue. Furthermore, degeneration might be present at midcarpal joint, the radiocarpal joint, or both. After removal of the necrotic lunate and the surrounding synovial tissue, saline irrigation was applied to remove the residual trivial pieces (Fig. 5A,B). Next, the proximal and distal articular surfaces of the lunate, the lunate facet of the distal radius, and the capitate were probed to see whether they had an intact subchondral bone or a floating articular. The arthroscopic classification of KD advocated by Bain and Begg^14^ was used in each case.

#### *Step 4: Fixation of the* *3D‐Printed Prosthesis*

**Figure 6:**
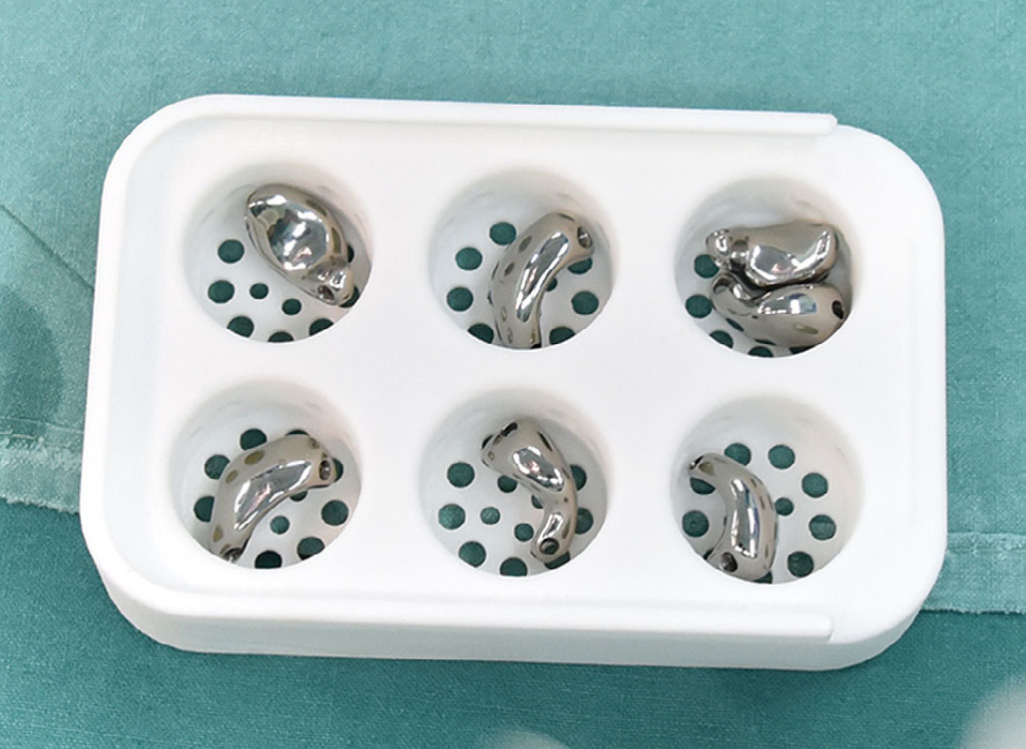
Intra‐operative view of three different sizes of 3D printing titanium lunate.

Afterwards, three different sizes of prosthesis were carefully inserted into the empty spaces (Fig. 6). This process was implemented by the forceps with rubber. Then, the wrist was flexed and extended passively to test which type of prosthesis fit the gap generated by excision of the necrotic lunate properly without subluxation and at suitable stress to the surrounding tissue and bone. During this process, we found out that prostheses made in ratios of 1:1 and 1:0.85 could be readily inserted into the gap, while it was difficult in the 1:1.1 prosthesis. Interestingly, impingement was noticed during all 1:1.1 prostheses and during part of 1:1 prostheses (three patients) tests, while subluxation of 0.85:1 prostheses were observed in two patients. Therefore, the 1:1 and 1:0.85 prostheses were both chosen as the final implant in different patients according to the intraoperative matching tests (Fig. 5C,D). Afterwards, the prosthesis was taken out and two resorbable sutures (#3) were threaded through the tunnels. The distal suture was passed through the volar ligament and fixed in the pull‐out manner, whereas the proximal suture was passed through the retinaculum before it was closed. If necessary, suture anchors were used for better prosthesis fixation. Intraoperative fluoroscopy was performed to confirm the lunate's position and carpal alignment.

#### *Step 5: Reconstruction*

Finally, the joint capsule and extensor retinaculum was repaired and the incision was primarily closed.

### *Postoperative Management*

The patients were postoperatively immobilized in a removable orthosis or plaster slab for 2 weeks. After removal, wrist exercises were started and continued for 4 to 16 weeks according to the patient's needs and progress.

### *Radiographic Evaluation*

After the surgery, all patients had radiographs (X‐ray, CT, and MRI) and received a clinical examination at the preselected time points.

### *Outcome Assessment*

A Jamar dynamometer (Sammons Preston Rolya, Bolingbrook, IL) was applied for measurement of the grip strength and a hand‐held goniometer (ACS90867, M&G, Shanghai, China) for range of movement (ROM; extension and flexion) with the healthy side selected for comparison.

### *Visual Analogue Scale (VAS)*

VAS is a psychometric scale that is generally used in pain scale surveys to understand varying degrees of pain experienced by a patient. It is also used in surveys to measure characteristics and attitudes across a range of continuous values; for example, if a person suffering from pain can rate that pain from a degree ranging continuously from “no pain” to “pain as bad as it could possibly be”. This scale was essentially developed to measure the basic element in continuity for pain measurement. For measurement of the magnitude of pain, the most used scale is “no pain” (corresponding to the scale of 0) and “pain too intense to be tolerated” (corresponding to the scale of 10). Respondents indicate their degree of acceptance to a statement by specifying a point on the continuous scale in between two endpoints. Intermediate numbers or pointers should be avoided in the VAS to avoid congregation. VAS was used to evaluate pain before surgery and at the time of final follow‐up.

### *Disabilities of the Arm, Shoulder, and Hand (DASH) Score*

The Disability of Arm, Shoulder, and Hand (DASH) questionnaire is a standardized measure which captures the patient’s own perspective of their upper extremity health status^15^. The DASH is a 30‐item disability/symptom scale examining the patient's symptoms, function, and sport. The items measure the degree of difficulty in performing different physical activities with the problem arm, shoulder, or hand; the severity of each of the symptoms of pain, activity‐related pain, tingling, weakness, and stiffness; and the problem's impact on social activities, work, sleep, and self‐image. Each item has five response options. To arrive at the DASH score, the scores for all of the items are then used to calculate a scale score ranging from 0 (no disability) to 100 (most severe disability)^16^. A total score >35 is considered a poor score, 15–35 is satisfactory, 6–15 is good, and 0–5 is excellent. At the final follow‐up, the DASH score were adopted to evaluate the treatment efficacy.

### *Mayo Modified Wrist Score (MMWS)*

MMWS is a clinician‐completed scoring system that is used to evaluate the level of disability in the wrist. The MMWS system mainly includes four aspects: pain, functional status (able to work), range of motion, and grip strength. The score standard had a maximum of 100 points (best possible outcome). A total score <60 is considered a poor score, 60–80 is satisfactory, 80–90 is good, and 90–100 is excellent. At the final follow‐up, MMWS were used to evaluate postoperative recovery of wrist function.

### *Patient Satisfaction*

Patient satisfaction is an important component of healthcare or treatment quality reflecting the healthcare or treatment provider's ability to meet patients’ needs and expectations. Our patient satisfaction was related to surgery and patient endorsements for the treatment effect. Patient satisfaction mainly includes five grades: very satisfied, satisfied, neutral (fair satisfied), dissatisfied, and very dissatisfied. The questions were measured on a five‐point scale from “very satisfied” to “very dissatisfied”. The patient chose relative point according to their own evaluation of treatment at last follow‐up.

### *Statistical Analysis*

All data were displayed as mean ± standard deviation (SD) with the statistical significance set at *P* < 0.05. The statistical analysis was performed using SPSS 17.0 software (SPSS Inc., Chicago, IL, USA). Statistical comparisons were performed with Student *t*‐test.

## Results

### *Follow‐up and General Results*

Patients were followed for 11–33 months, with an average follow‐up duration of 19.4 months. The mean operation time (range, 45 to 56 min) was 51.8 ± 4.44 min (Table 1).

### *Radiographic Evaluation*

**Figure 7:**
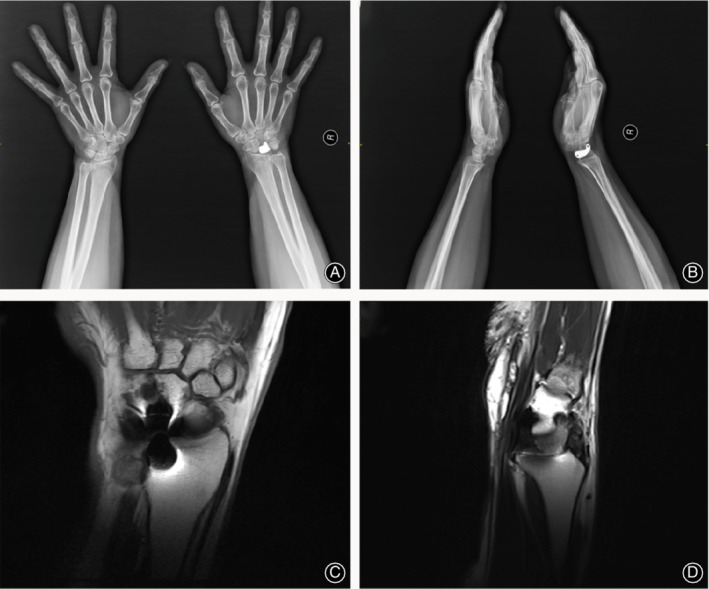
Views of the position of the 3D printing lunate and articular surface of radium from Fig. 1 at 30‐month follow‐up. (A, B) (X‐ray) show no dislocation nor subluxation of the prosthesis; (C, D) (MRI) show no degenerative change observed on the articular surface of the radium.

**Figure 8:**
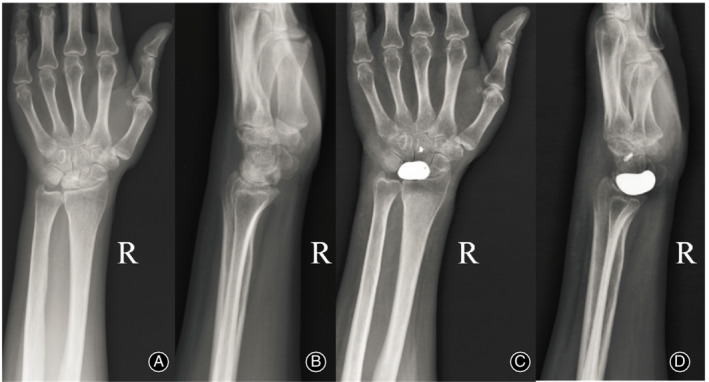
Radiologic data of 46‐year‐old female patient. (A, B) preoperative anteroposterior/ lateral wrist X‐rays; (C, D) postoperative anteroposterior/ lateral wrist X‐rays at the last follow‐up.

**Figure 9:**
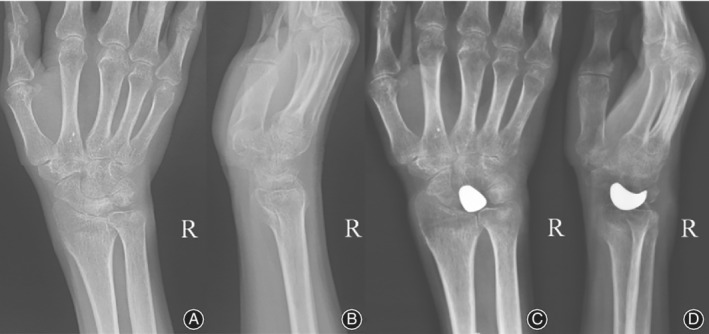
Radiologic data of 53‐year ‐old male patient. (A, B) preoperative anteroposterior/ lateral wrist X‐rays; (C, D) postoperative anteroposterior/ lateral wrist X‐rays at the last follow‐up.

The imaging examination showed that the implant prosthesis remained in position and did not change over time. Neither dislocation nor subluxation was observed at the end of follow‐up (Figs 7, 8, 9).

### *Outcome Assessment*

The mean extension range of the wrist significantly increased from preoperative 44° ± 9.6° to postoperative 60° ± 3.5°(*P* = 0.012 < 0.05). The mean flexion range of the wrist significantly increased from preoperative 40° ± 10.6° to postoperative 51° ± 6.5° (*P* = 0.04 < 0.05). The grip strength of the affected side compared with the unaffected side improved from 47% ± 7.8% to 88.6% ± 5.9%.

### *VAS*

The VAS for wrist pain was 7.3 ± 1.2 preoperatively and 0.2 ± 0.5 at the last follow‐up (*P* = 0.0001 < 0.05). Furthermore, four of the five patients were free from pain and the remaining patients had mild wrist pain during strenuous activity.

### *DASH* *Score*

The mean DASH score was 10 ± 2.8 at the last follow‐up.

### *MMWS*

The average MMWS was 79 ± 7.4 at the last follow‐up.

### *Patient Satisfaction*

At the last follow‐up, three patients had resumed their work at the same position and two had changed positions. Patients were either very satisfied (three patients), satisfied (one patient), or neutral (one patient) with the surgical outcome. The treatment outcomes were list in Table 2.

**Table 2:** The preoperative and postoperative evaluation of the patients

Case	Pain on VAS		Mayo modified wrist score		DASH score	Patient satisfaction
	Preoperation	Postoperation				
1	6	0		75	11.2	Satisfied
2	8	1		70	14.2	Neutral
3	6.5	0		90	7.8	Very satisfied
4	7	0		80	9.6	Very satisfied
5	9	0		80	7.2	Very satisfied
Mean	7.3	0.2		79	10	

### *Intraoperative Difficulties*

It was difficult to suture prosthesis with surrounding tissue during operation. Prosthesis fixation was difficult and important for preventing postoperative subluxation or dislocation.

### *Complications*

There were no incision infections and the wounds healed well. No cysts or synovitis in the radial or carpal bones were observed. Osteoarthritis was observed in two patients before surgery. Among them, no significant aggravation in arthritis was observed compared with the pre‐operative manifestation. As for the patients at grade 3, arthritis was partially reversed.

## Discussion

### *KD* *Treatment*

KD is characterized by ischemic necrosis of the lunate and often results in a predictable pattern of carpal change and degeneration and lunate collapse. The goals of pain relief, motion preservation, strength maintenance, and good functional outcomes are essential for successful treatment. Various therapeutic proposals, including lunate revascularization^17^, radial or ulnar wedge and osteotomies^18^, ^19^, limited intercarpal fusions^20^, and excision arthroplasty^17^, have been advocated as treatments for advanced KD. However, there is no procedure that consistently and reliably achieves these outcomes. With the advancements being made in understanding the cause and pathophysiology of KD, it has been recognized that the preferred method should be individualized based on the patient's age, expectations, and stage of KD. The 3D‐printed lunate is an ideal alternative for individualized reconstruction of the collapsed lunate, which was not only able to restore the anatomical structure of the carpal, prevent capitate collapse, and correct the radioscaphoid alignment, but also preserve wrist mobility along with pain relief. In the present study, the patients showed significant functional improvement and pain relief at the end of the follow‐up period. Considering that the patients in our study were quite young, all of them expressed their satisfaction after they returned to work. These results showed that 3D‐printed lunate prosthesis may be a good alternative for advanced KD.

In case of a completely necrotic lunate bone, revascularization procedures are unlikely to be the optimal treatments, whereas salvage options such as proximal row carpectomy and total wrist fusion may lead to degenerative arthritis resulting from the abnormal loading at the radiocarpal or midcarpal articulation, and are typically reserved for older patients with lower functional demands^5^, ^14^. The techniques for lunate excision and interposition have evolved over time since starting with vitallium prostheses as described by Lippman in 1949^9^, and followed by several alternatives such as silastic and pyrocarbon prostheses. Recently, interposition of tendon‐ball implants or fascia, and autografts, such as pedicled pisiform, have also had their advocates^21^, ^22^. These surgical options are beneficial in preserving the ROM of the wrist. However, they may also be potentially associated with disease progression such as loss of carpal height or inherent instability^5^. For example, it is common to see calcification or ossification in the defect filled by the tendon ball, whereas subluxation, dislocation, or impaction of the prosthesis may be associated with silicone or pyrocarbon prostheses^23^. In our study, by the end of the follow‐up period, the implant was well‐tolerated clinically and radiographically. The wrists remained correctly aligned without any anterior or posterior subluxation. Several reasons may account for this good outcome. First, the 3D‐printed lunate resembled the exact morphology of the impaired lunate, which is unachievable by other fabricating techniques due to its irregular shape. After the replacement, anatomical stability was restored. Meanwhile, the size of the lunate is much smaller than the pyrocarbon, therefore, the capsule incision is quite small and there is no need for partial resection of the surrounding bone structures. Furthermore, the tunnels designed into the prosthesis allow for the passage of resorbable sutures, which are helpful for the transit fixation of the prosthesis before the healing of the capsule; thus, patients could start performing their rehabilitation exercises immediately after the surgery. In our experience, the tunnels made on the lunate prosthesis is helpful to avoid stiffness by allowing the movement of wrist after the operation immediately and preventing the dislocation of the implant. Furthermore, our results demonstrated that the ideal size of the lunate prothesis varies in different patients, and the final choice depends on the intra‐operative exploration.

### *Lunate Excision and* *3D‐Printed Lunate Prosthetic Arthroplasty*

The surgical process for arthroplasty using a 3D‐printed lunate is quite simple with only two steps: excision of the lunate and insertion of the prosthesis. Despite few reports comparing the surgical duration of different procedures, the mean operative time in our series was 51.8 min, which is relatively short for KD procedures. For other arthroplasty procedures, partial radial styloidectomy and partial resection of the scaphoid and the capitates are routinely required. Meanwhile, in these procedures, to facilitate the insertion of prosthesis and improve the postoperative ROM of the wrist, surgical procedures such as deepening the radial glenoid concavity or capsular plication may also be needed^23^. These manipulations are time‐consuming and greatly depend on the surgeon's individual experience, which discourages their adoption by young surgeons. In our study, the learning curve of arthroplasty is quite simplified. Meanwhile, when data collection and 3D reconstruction are complete, surgeons can formulate a plan and rehearse the operations before the actual surgery, which could improve the safety and efficiency of the operation.

### *Prevention of Complications*

The prevention of synovitis, arthritis, or cyst formation by the prosthesis is a major factor in the success of arthroplasty^24^. All of the patients in our study underwent MRI analysis at the last follow‐up and no cysts, synovitis, or osteoarthritic progression were found. This may be due to the following reasons. First, as the shape of lunate is irregular and the gap created by the removal of the necrotic lunate is limited, these factors compounded the difficulties of prosthesis insertion. Thus, the susceptibility of the surface of the prosthesis to microwear and the formation of metal debris are increased. The holes specially designed in the prosthesis for clamping by the forceps facilitated insertion in our study. Second, the rubber placed on the forceps avoids damage to the prosthesis and maintains its integrity. Third, titanium is the most frequently used material for the fabrication of arthroplasty prosthesis due to its high stability^24^. Its degradability is much lower than other elements, such as silicone. These factors may account for the rare incidence of synovitis in our study.

### *Disease Classification*

A disease classification scheme is good for as long as it can direct the definitive procedure to be suggested in a large variety of clinical circumstances. The classification for KD devised in 1977 and modified in 1993 by Lichtman is based on the osseous classification and is the most widely used to guide treatment at various stages of the disease^25^, ^26^. In 2010, a stage IIIC disease category referring to a coronal fracture or fragmentation of the lunate was added to the staging system. In addition to the radiologic osseous classification system, Bain and Begg^14^ proposed an arthroscopic method to assess and classify KD. They defined an articular surface as nonfunctional if there was extensive fibrillation, fissuring, extensive articular loss, a floating articular surface, fracture, or arthritis. The grading system ranges from grade 0 to 4 in accordance with the number of nonfunctional articular surfaces. In our study, there was one Lichtman IIIA case (grade 2a), two Lichtman IIIB cases (one grade 2a and one grade 3), one Lichtman IIIC case (grade 2b), and one Lichtman IV case (grade 4). There was an overlap between the stages of the two different classification systems. As the severity of the articular involvement is often underestimated when evaluated just by plain radiographs^14^, and it is a challenge for surgeons to distinguish Lichtman grades IIIA from IIIB^27^, a combination of Lichtman's classification with the arthroscopic classification is beneficial for preoperative assessment and deciding the optimal surgical approach. In our study, the treatment efficacy for the patients in grade 2 was most promising, without degenerative osteoarthritis at the latest follow‐up visits. While for patients in grades 3 and 4, the progression of KD was halted and even partially reversed. To optimize the treatment efficacy and avoid complications, a timely and comprehensive evaluation is vital.

### *Limitation of the Study*

Although our initial results are promising, there are several limitations and unanswered questions. One limitation is that our study has a short follow‐up period and the number of patients in the series is limited. Clinical and radiological studies of larger cohorts with longer follow‐up durations are warranted to determine the advantages of 3D‐printed implants for treatment of advanced KD and its specific indications in terms of different disease stage and classification. In addition, the 3D‐printed lunate is costlier than routine surgery. Therefore, epidemiological data about the long‐term costs of different interventions, taking into account complications and treatment effects, are needed. Nevertheless, our study demonstrated that arthroplasty using a personalized 3D‐printed titanium lunate provides a completely novel option for the treatment of advanced KD.

### *Conclusions*

For patients suffering advanced Kienböck's disease, lunate excision followed by 3D‐printed prosthetic arthroplasty can reconstruct the anatomical structure of the carpal tunnel, alleviate pain, and improve wrist movement. The current study provides evidence that arthroplasty using a personalized 3D‐printed titanium lunate is a completely novel option for the treatment of advanced KD and this cutting‐edge technology may be employed to improve outcomes in this difficult group of patients. Meanwhile, this study may be beneficial for the accumulation and spread of experience for designing and fabricating the individualized titanium lunate. Further research on the feasibility of this new approach on a large scale with a long follow‐up are warranted.

## Funding

The study was supported by National Key R&D Program of China (2018YFC2002303, 2018YFB1105600, 2018YFC2001300, 2018YFA0703000, 2018YFB1107000) and National Nature Science Foundation of China (81572156, 81772326), China Postdoctoral Science Foundation (2018M630451, 2018M642042, 2018M640406), National Postdoctoral Program for Innovative Talents (BX20180194), Project of Shanghai Jiading National Health and Family Planning Commission (KYXM 2018‐KY‐03), Project of Shanghai Clinical Medical Center (2017ZZ01023), Shanghai Municipal Key Clinical Specialty.

## Conflict of Interest

All authors declare that they have no conflict of interest.
